# Impact of Acoustic and Interactive Disruptive Factors during Robot-Assisted Surgery—A Virtual Surgical Training Model

**DOI:** 10.3390/s20205891

**Published:** 2020-10-17

**Authors:** Magret Krüger, Johannes Ackermann, Daniar Osmonov, Veronika Günther, Dirk Bauerschlag, Johannes Hensler, Jan-Hendrik Egberts, Sebastian Lippross, Georgios Gitas, Thomas Becker, Nicolai Maass, Klaus-Peter Jünemann, Ibrahim Alkatout

**Affiliations:** 1 Department of Obstetrics and Gynecology, University Hospitals Schleswig-Holstein, Campus Kiel, Arnold-Heller-Str. 3 (House C), 24105 Kiel, Germany; magret.krueger@uksh.de (M.K.); johannes.ackermann@uksh.de (J.A.); veronika.guenther@uksh.de (V.G.); dirk.bauerschlag@uksh.de (D.B.); nicolai.maass@uksh.de (N.M.); 2 Department of Urology and Pediatric Urology, University Hospitals Schleswig-Holstein, Campus Kiel, Arnold-Heller-Str. 3 (House C), 24105 Kiel, Germany; Daniar.Osmonov@uksh.de (D.O.); klaus-peter.juenemann@uksh.de (K.-P.J.); 3 Department of Radiology and Neuroradiology, University Hospitals Schleswig-Holstein, Campus Kiel, Arnold-Heller-Str.3 (House D), 24105 Kiel, Germany; Johannes.hensler@uksh.de; 4 Department of General, Visceral, Thoracic, Transplant, and Pediatric Surgery, University Hospitals Schleswig-Holstein, Campus Kiel, Arnold-Heller-Str. 3 (House C), 24105 Kiel, Germany; jan-hendrik.egberts@uksh.de (J.-H.E.); sekretariat.profbecker@uksh.de (T.B.); 5 Department of Trauma Surgery and Orthopedics, University Hospitals Schleswig-Holstein, Campus Kiel, Arnold-Heller-Str. 3 (House C), 24105 Kiel, Germany; Sebastian.lipross@uksh.de; 6 Department of Obstetrics and Gynecology, University Hospitals Schleswig-Holstein, Campus Lübeck, Ratzeburger Allee 160 (House A), 23538 Lübeck, Germany; georgios.gitas@uksh.de

**Keywords:** laparoscopy, virtual reality trainer, robotic surgery, complications, surgical education, disruption during surgery

## Abstract

The use of virtual reality trainers for teaching minimally invasive surgical techniques has been established for a long time in conventional laparoscopy as well as robotic surgery. The aim of the present study was to evaluate the impact of reproducible disruptive factors on the surgeon’s work. In a cross-sectional investigation, surgeons were tested with regard to the impact of different disruptive factors when doing exercises on a robotic-surgery simulator (Mimic Flex VR^TM^). Additionally, we collected data about the participants’ professional experience, gender, age, expertise in playing an instrument, and expertise in playing video games. The data were collected during DRUS 2019 (Symposium of the German Society for Robot-assisted Urology). Forty-two surgeons attending DRUS 2019 were asked to participate in a virtual robotic stress training unit. The surgeons worked in various specialties (visceral surgery, gynecology, and urology) and had different levels of expertise. The time taken to complete the exercise (TTCE), the final score (FSC), and blood loss (BL) were measured. In the basic exercise with an interactive disruption, TTCE was significantly longer (*p* < 0.01) and FSC significantly lower (*p* < 0.05). No significant difference in TTCE, FSC, or BL was noted in the advanced exercise with acoustic disruption. Performance during disruption was not dependent on the level of surgical experience, gender, age, expertise in playing an instrument, or playing video games. A positive correlation was registered between self-estimation and surgical experience. Interactive disruptions have a greater impact on the performance of a surgeon than acoustic ones. Disruption affects the performance of experienced as well as inexperienced surgeons. Disruption in daily surgery should be evaluated and minimized in the interest of the patient’s safety.

## 1. Introduction

Virtual reality trainers have become an essential part of training in minimally invasive surgery. The impact of virtual reality trainers on surgical performance has been confirmed in several studies [1,2,3].

Disruption during surgery has become a widespread problem in operating rooms. The most common and severe disruptive factors in operating rooms were analyzed in a review of 17 studies; movement in the operating room and case-foreign communication were identified as the most common factors [4]. The most severe disruptions were due to dysfunctional equipment and procedural deviation [4]. The consequences include longer operating times due to slower motion, errors [5], and more frequent surgical site infections [4].

Individual disruptive factors are known to have different impacts. Acoustic and interactive disruptions were reported to have adverse effects on the performance of surgeons [6,7,8,9,10]. Although the negative effect of cognitive distraction on surgical outcome is well known and has been proven in numerous studies, diverse data have been reported for acoustic disruptions in the operating room [5,6,11]. Music, for instance, may have a positive or negative effect, depending on the genre [8,12]. Acoustic disturbance is known to have a greater impact than visual disturbance [4]. Therefore, two disruptive factors were selected for the present study: One was acoustic and the other interactive.

Some studies have shown that persons who play video games in their personal lives are able to handle virtual reality trainers more competently [13,14,15,16]. Medina and Barraza found that professional musicians are able to control their attention more effectively than nonmusicians [17]. Moglia et al. noted that playing video games and practicing music were associated with the highest locomotor skills on a virtual reality trainer for robot-assisted surgery [18]. However, other studies did not confirm these correlations [13,14,19]. The two activities, i.e., playing video games and practicing music, may exert an impact on a surgeon’s concentration abilities.

A few studies have demonstrated the negative impact of disruptive factors on the surgical performance of students and surgical novices practicing on a virtual reality trainer [5]. Experienced surgeons performing conventional surgery have been tested for the impact of disruptive factors [9,20,21,22]. However, data concerning the impact of distractions on surgical performance among experienced surgeons performing minimally invasive surgery are scarce [22]. To the best of our knowledge, we lack any comparison of the impact of distractions on the surgical performance of experts and novices on virtual reality trainers, robotic surgery, or laparoscopic surgery. Due to their high self-esteem, experienced surgeons—in contrast to novices—are more likely to handle potential disruptions casually during surgery [20]. Based on organizational structures, clinics rely on a surgeon’s accessibility even during operations and thus accept a potentially higher risk of complications. In the interest of the patient’s safety, it would be appropriate to investigate the actual difference between experts and novices in regard of their surgical performance during disruption, as well as the impact of their self-estimation.

However, we lack a common definition for the terms “expert” and “beginner.” As a result, different methods of classification have been used. Distinctions have been made on the basis of professional experience in years, number of operations, or even self-assessment [22,23,24,25,26,27]. Specific conditions and the numbers of subgroups in the individual studies vary widely. Besides, surgical societies have diverse requirements for obtaining specialist certificates [28,29,30].

The aim of the present study was to investigate the impact of an interactive and a solely acoustic disturbance while surgeons use a virtual reality trainer. We focused on the experience of the surgeons and their skills in practicing music and playing video games. In addition, the division of surgeons into experts and novices was evaluated on the basis of their self-assessment and professional experience.

## 2. Materials and Methods

The investigation was performed at the 11th Symposium of the German Society for Robot-Associated Urology (Deutsche Gesellschaft für Roboter-assoziierte Urologie e.V., DRUS). As the symposium attracted surgeons with different levels of experience, DRUS 2019 appeared to be the perfect setting for the present investigation.

The attendees of DRUS 2019 were requested to participate in virtual robotic stress training. As the level of experience was one of the issues addressed in the study, participants with different levels of experience were enrolled. Data concerning their level of experience were collected on questionnaires to be filled during the exercises. Written informed consent was provided by every participant on the questionnaire.

**Figure 1:**
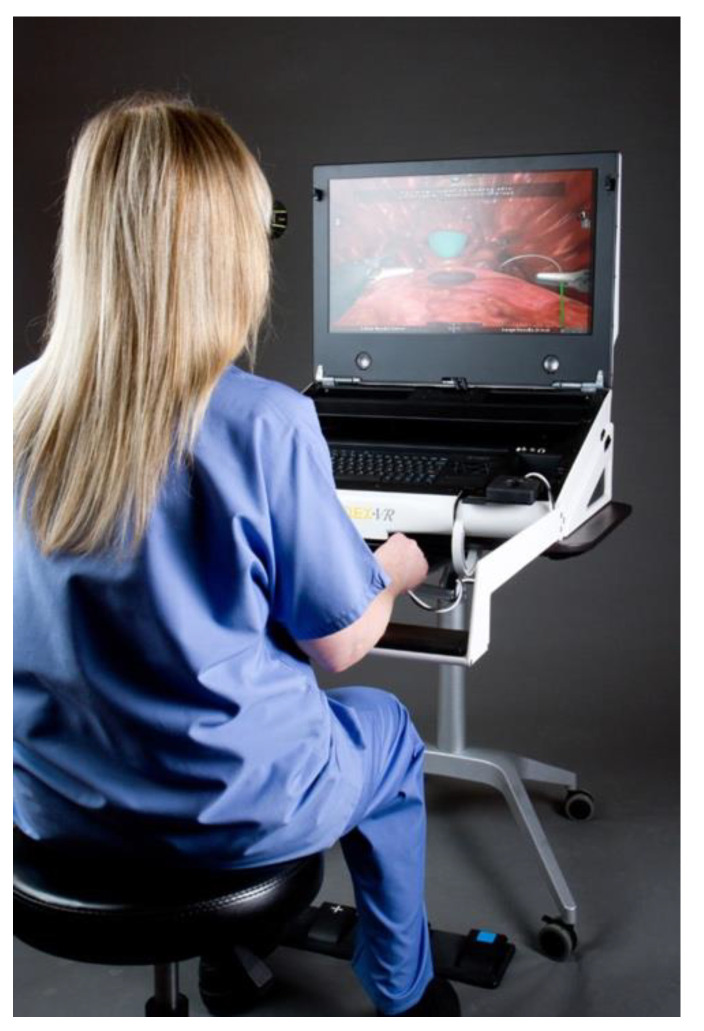
Mimic Flex VR^TM^ in use (obtained with the kind permission of Mimic Simulation).

The tasks were performed on the Mimic Flex VR^TM^ (811 First Ave, Suite 408, Seattle, WA 98104 USA), which was a virtual reality trainer presented at the industrial exhibition during DRUS 2019. The Mimic Flex VR^TM^ is compact and portable. The surgeon sits in front of a 3D screen and performs the tasks with two controllers, similar to controllers in the operating room (Figure 1). The participants were required to perform two tasks of different levels of difficulty.

**Figure 2:**
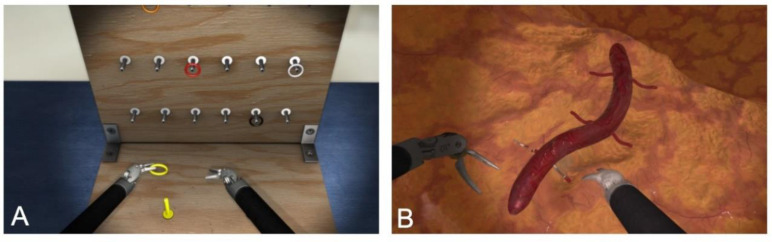
(**A**) Set up for Peg Board and (**B**) for Energy Dissection (pictures from the simulator system of Mimic Flex VR^TM^ obtained with the kind permission of Mimic Simulation).

In the first exercise (Peg Board), a ring had to be removed from a stick on the ground with one hand using an instrument, taken over by the other hand with the aid of an instrument, and placed on another stick on the wall, as shown in Figure 2a. The challenge was to transfer the ring from one position to another with the aid of instruments.

In the second exercise (Energy Dissection), six vessels derived from one large vessel had to be coagulated and then cut off, as shown in Figure 2b. Energy Dissection required two types of instruments, namely, one for coagulation and the other for cutting. The aim was to coagulate the vessel and then cut it off without bleeding. The grade of coagulation had to be perfect, failing which the coagulated vessel would bleed or be ruptured. After a certain period of time, the vessels started to bleed again. The exercise was completed when all six vessels had been cut off and all bleeding had ceased (Figure 2).

The camera had to be moved in both exercises. Peg Board was estimated to be the easier of the two tasks.

To investigate the impact of disruptive factors, the participants were exposed to two different stimuli. The disruption during Peg Board was interactive. The participants were asked to complete a questionnaire. The majority of the questions called for an oral answer. For four questions, the participants were asked to look away from the simulator and rate their surgical experience (as surgeon and as assistant), musical activity, and playing video games, on a visual analog scale (VAS) from 0% to 100%. The questionnaire included questions about gender, age, professional and surgical experience, performance of music, and playing video games. The questionnaire was provided in German (attached in Supplementary data in German and English).

The questionnaire included questions about gender, age, surgical experience, performance of music, and playing video games (see Supplementary data). The disruption during Energy Dissection was solely acoustic. The participants were made to wear noise-canceling headphones and were exposed to a soundtrack starting with a sinus rhythm followed by an asystole, hectic electronic music, and simultaneously an interview with a German soccer trainer (Thomas Tuchel).

**Figure 3:**
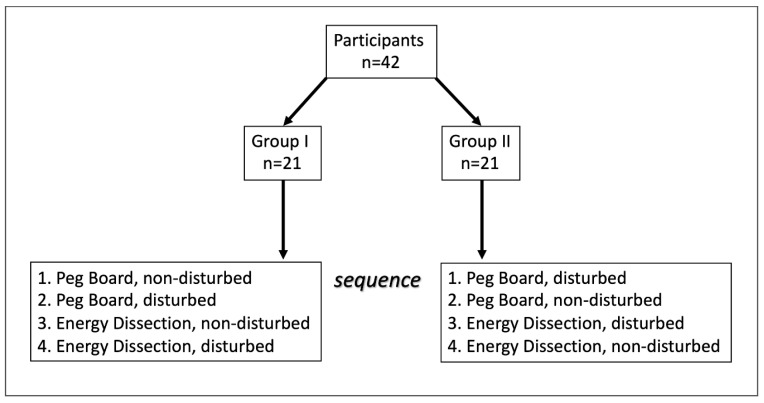
Test sequence.

To avoid a potential bias due to habituation, the participants were divided into two groups alternately. Both groups started with two attempts of Peg Board, followed by two attempts of Energy Dissection. Group I performed the task without disruption and then with the disruptive factor, whereas Group II performed the tasks in the opposite sequence (Figure 3). The final score in points, the time to complete exercise, and blood loss during Energy Dissection were measured and evaluated.

We selected the time taken to complete the exercise (TTCE) and blood loss during Energy Dissection (BL) as parameters of patient safety and the final score (FSC) as a measure of general performance. The final score (FSC) was calculated with the MScore^®^ scoring system developed by Mimic Simulation, based on the fundamentals of laparoscopic surgery (FLS) endorsed by the American College of Surgeons [31]. FSC was derived from other parameters such as economy of motion, blood loss, broken vessels, the number of times an object was dropped, excessive force of instruments, collision of instruments, instruments out of view, master workspace range, and misapplied energy. Similar scores have been used by other authors for investigating surgical education on virtual reality trainers [3,18].

The results of the disrupted (D) and nondisrupted (ND) tasks on the virtual reality trainer were compared. Overall differences (∆) in the final score (FSC), the time taken to complete the exercise (TTCE), and blood loss during Energy Dissection (BL) were calculated for the disrupted and nondisrupted tasks and correlated with factors such as gender, age, professional experience, playing an instrument, and playing video games.

The surgeons’ self-estimation of their expertise in minimally invasive surgery was investigated via the questionnaire and then correlated with the number of minimally invasive operations performed, experience in minimal invasive surgery in years, and professional experience in years.

Data for the performance parameters were collected by the simulator software of Mimic Flex VR^TM^. The personal data of the participants were matched using Microsoft Excel and analyzed with SPSS. Variables were checked for normal distribution with the Kolmogorov-Smirnov test. Wilcoxon’s test for paired differences was used for non-normally distributed data. The Mann-Whitney U test was used for pairwise comparisons without normal distribution. Spearman’s rho test was used to analyze correlations of two variables. VAS scores were described as follows: <20, very low; 20 to <40, low; 40 to <60, moderate; 60 to <80, high; and 80 to 100, very high. Spearman’s rho test was used for correlation analysis when significant deviations from normal distribution were found. The correlation coefficient (R) was evaluated as follows: R ≤ 0.2, no correlation; 0.2 < R ≤ 0.5, weak to moderate correlation; 0.5 < R ≤ 0.8, strong correlation; and 0.8 < R ≤ 1.0, very strong correlation. Tests were performed bilaterally and a significance level of 5% was used (*p* < 0.05). Statistical analysis was performed by a professional medical statistics company (Medistat GmbH, Kiel, Germany).

## 3. Results

Forty-two persons were included in a cross-sectional study on the virtual reality trainer Mimic Flex VR^TM^. The mean age of the participants was 38 years (range: 24–80 years). The majority were male (30 men and 12 women; 71.5% versus 29%). The median professional experience of the participants was 9.5 years, but the duration of their experience in minimally invasive surgery was only 4.5 years. The median number of minimally invasive operations performed until the study was 23 (range: 0–2500 operations). In the self-assessment, the median rating for experience in minimally invasive surgery was 23%, and the interquartile range was 0–84%. Experience in practicing music and playing video games were also in the lower median range (26% and 28%). The results of the survey regarding the experience of the participants are shown in Table 1.

**Figure 4:**
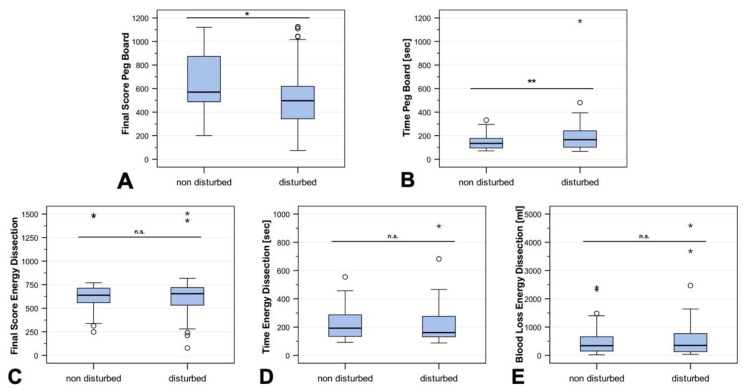
Comparison of the final score (FSC) and the time to complete the exercise (TTCE) for Peg Board (**A**) and (**B**) and Energy Dissection (**C**) and (**D**) with and without disturbance. (**E**) Blood loss in Energy Dissection with and without disturbance. The Peg Board task yielded a higher final score (*p* < 0.05) and was completed in a shorter period of time (*p* < 0.01) without disturbance, whereas Energy Dissection revealed no significant difference between the attempts. Small circles and stars show outliers.

Of the 42 participants who performed the exercises, all completed Peg Board (disturbed and nondisturbed), and 38 completed both attempts at Peg Board and both attempts at Energy Dissection. As there were many statistical outliers in both directions, the results were not normally distributed. Therefore, medians are given as descriptive information. The results of the Peg Board (interactive disruption) exercise differed significantly between the two groups (disturbed vs. non-disturbed). The final score (FSC) in the disturbed group was significantly lower (median 496 vs. 570, *p* < 0.05), and the time to complete the exercise (TTCE) significantly longer (median 165 vs. 134 s, *p* < 0.01). In contrast, the results of the Energy Dissection (acoustic disruption) exercise did not differ significantly between the two groups in terms of the final score (FSC) (median 650 vs. 635, *p* = 0.91), time to complete the exercise (TTCE) (median 164 vs. 194 s, *p* = 0.79), and blood loss (median 364 vs. 345 mL, *p* = 0.24). The distributions of these exercises are shown in Figure 4.

To investigate the influence of gender on the participants’ susceptibility to disturbance, we performed a gender-specific analysis of differences (∆, disrupted—nondisrupted) in the final score (FSC), time to complete the exercise (TTCE) for Peg Board and Energy Dissection, as well as for blood loss due to energy dissection. No gender-specific difference was registered for either of the exercises.

To investigate the influence of age, professional experience, the number of minimally invasive surgeries, experience in practicing music, and experience in video games on susceptibility to disturbance, we performed a subgroup and correlation analysis between groups and differences (∆ of disrupted—nondisrupted) in the final score (FSC), and time to complete the exercise (TTCE) for Peg Board and Energy Dissection, as well as for blood loss during energy dissection.

**Figure 5:**
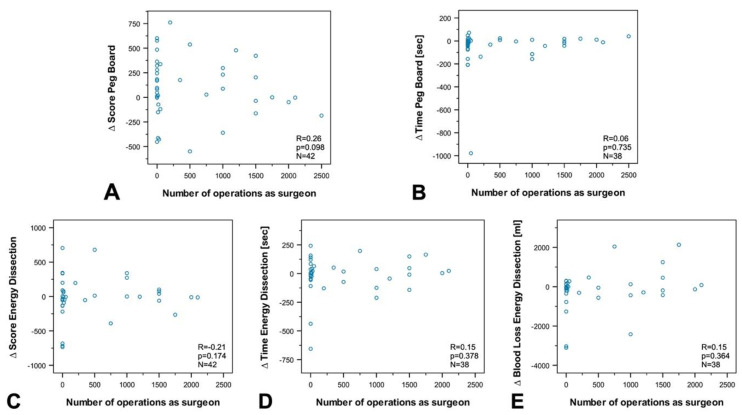
Correlation analysis between the number of minimally invasive operations as a surgeon and (**A**) ∆ Final Score in Peg Board, (**B**) ∆ Time to complete Peg Board, (**C**) Final Score Energy Dissection, (**D**) ∆ Time to complete Energy Dissection, and (**E**) ∆ Blood Loss Energy Dissection. No significant correlation was registered for any of the values (correlation coefficient: R, statistical significance: p, number: N).

**Figure 6:**
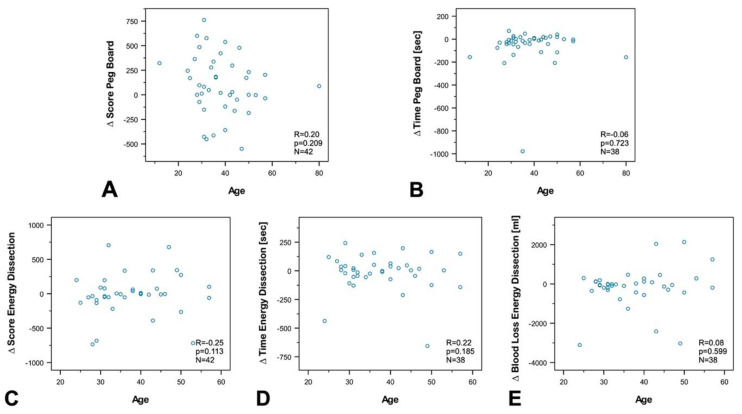
Correlation analysis between the age of the participants and (**A**) ∆ Final Score in Peg Board, (**B**) ∆ Time to complete Peg Board, (**C**) Final Score Energy Dissection, (**D**) ∆ Time to complete Energy Dissection, and (**E**) ∆ Blood Loss Energy Dissection. No significant correlation was found for any of the values (correlation coefficient: R, statistical significance: p, number: N).

**Figure 7:**
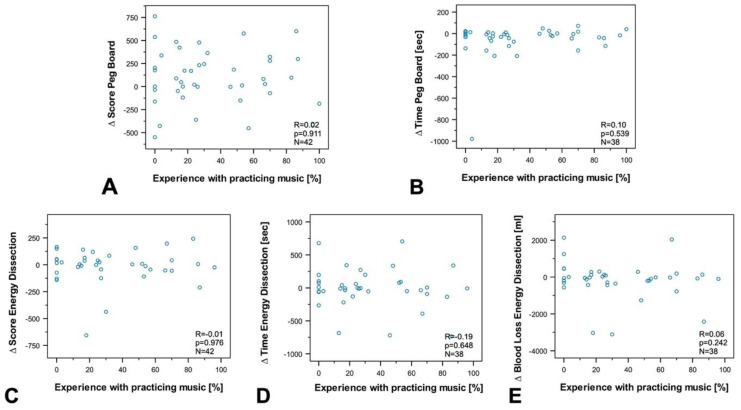
Correlation analysis between experience in practicing music, and (**A**) ∆ Final Score in Peg Board, (**B**) ∆ Time to complete Peg Board, (**C**) Final Score Energy Dissection, (**D**) ∆ Time to complete Energy Dissection, and (**E**) ∆ Blood Loss Energy Dissection. No significant correlation was found for any of the values (correlation coefficient: R, statistical significance: p, number: N).

**Figure 8:**
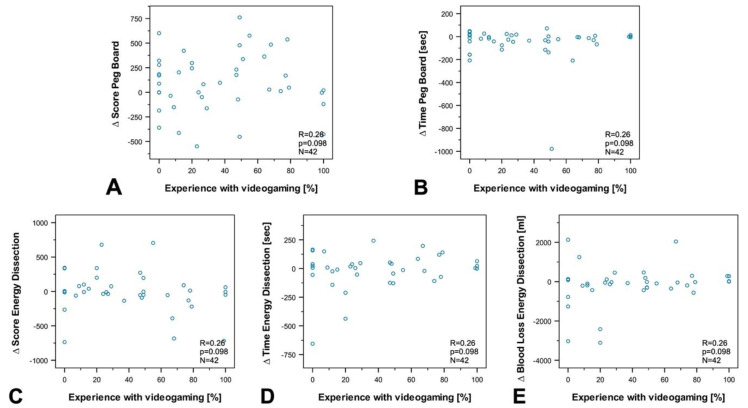
Correlation analysis between experience in video games, and (**A**) ∆ Final Score in Peg Board (**B**) ∆ Time to complete Peg Board, (**C**) Final Score Energy Dissection, (**D**) ∆ Time to complete Energy Dissection, and (**E**) ∆ Blood Loss Energy Dissection. No significant correlation was registered for any of the values (correlation coefficient: R, statistical significance: p, number: N).

The analyses revealed no correlation between age (Figure 5), experience in minimally invasive surgery (Figure 6), experience in practicing music (Figure 7), experience in video games (Figure 8), and the results of the exercises. Furthermore, professional experience in years, experience in minimally invasive surgery in years, and self-assessment in minimally invasive surgery had no significant impact on the outcome of the exercises. Since none of the abovementioned factors influenced the results of the exercise or the participants’ susceptibility to disturbance, we performed no further subgroup analysis of experience in practicing music or experience in video games, and also dispensed with a division of participants into experts and novices.

To examine the correlation between the participants’ self-assessment and objective professional experience, we performed correlation analyses between self-estimated expertise and the number of minimally invasive surgeries, as well as experience in minimally invasive surgery in years and professional experience in years. A very strong positive correlation was registered between self-assessment and the number of minimally invasive operations performed (correlation coefficient (R) 0.93; *p* < 0.001). A strong positive correlation was also found in regard of professional experience and experience in minimally invasive surgery in years (correlation coefficient (R) 0.76 and 0.79; *p* < 0.001). Thus, this correlation was lower than that observed for the number of minimally invasive surgeries.

**Figure 9:**
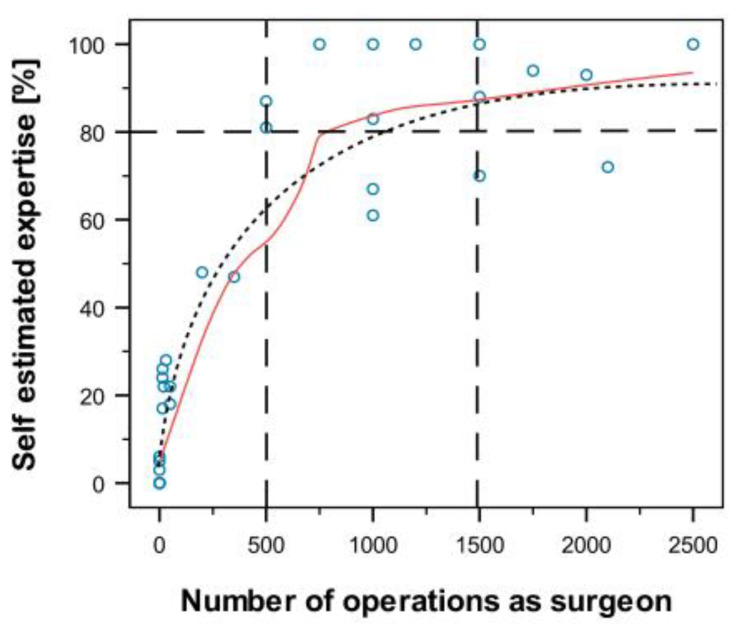
Correlation between self-estimated expertise as a surgeon and the number of minimally invasive operations performed as a surgeon. Due to a nonlinear correlation, a Loess fitting curve (85% of the values) was calculated. This curve approximates a logarithmic curve (see curved dotted line). If this curve is used as a basis and interindividual scattering is suspected, a range of 500–1500 (see vertical broken lines) operations may be used as a prerequisite for an expert (limit 80%, see horizontal broken line).

The correlation between self-assessment and the number of minimally invasive surgeries was not linear. Therefore, a Loess fitting curve (85% of the values) was calculated (see Figure 9). Based on a classical learning curve, this adaptation curve cuts the threshold value of 80% for a very high self-assessment of one’s own expertise for about 1000 surgeries. In the event of uncertain data and individual differences in the learning curve, a range of 500–1500 operations was identified as the potential criterion of an “experienced” surgeon (see Figure 9).

## 4. Discussion

In the present study, we registered different effects of disruption on performance during surgical training on a virtual reality trainer. Interactive disruptions appear to have a greater negative impact on the outcome of surgical procedures in respect of quality and time compared to acoustic disruptions. To the best of our knowledge, no previous study has been focused on a direct comparison of acoustic disruption and disruption involving mental activity.

The negative effect of cognitive distraction on surgical outcome has been reported in numerous studies and was confirmed by our work [5,6,11]. Diverse data have been reported about acoustic disruptions in the operating room. Generally, acoustic disruption is divided into case-foreign conversation or loud machinery on the one hand and music on the other. Noise is known to distract surgeons [7,9,10], whereas music can be distractive but also supportive [8,12]. We used a mixture of both in the form of electronic music, abnormal electrocardiographic sounds, and an interview.

Unspecific noise levels in the operating room, including conversation, music, and noise from machines and equipment are correlated with more numerous surgical site infections [9]. In contrast, Moorthy et al. [20] registered no change in the performance of surgeons during a laparoscopic task when being exposed to an acoustic disturbance. In fact, an improvement in surgical performance was observed when the subjects listened to rhythmic music while performing tasks on a DaVinci trainer [8]. Thus, not every noise in the operating room is a disruptive factor that would impair a surgeon’s performance. However, any noise could become disturbing and should be taken into account in any operation. In contrast, interactive disruptions are always a significant disruptive factor that endangers patient health and safety. This should be taken into consideration and prevented if possible. All persons in the operating room should refrain from interactive disruption. This also applies to other forms of training such as live surgery events or when teaching surgery to medical students [32].

In view of the fact that our participants fared significantly worse when disturbed in Peg Board than during Energy Dissection, the two levels of difficulty must be taken into account. As described earlier, we consider Energy Dissection the more difficult task because of its complex requirements (exact grade of anticoagulation, vessels starting to bleed again after a certain time, instruments with different functions, and longer time to complete the exercise). The participants appear to have blocked disruption and performed better during this more difficult task. This is indicative of better resistance to disruption in a state of intense concentration. On the other hand, Siu et al. registered a more pronounced negative effect of the same disruptive factor when performing difficult tasks on a DaVinci simulator [7]. Another potential confounder is the fact that Energy Dissection was always performed as the second task. Although we randomized the order of disruption and no disruption, we did not randomize the sequence of the exercises.

Our data showed that performance during distraction was independent of the surgeons’ level of experience. Studies analyzing the performance of expert and novice surgeons under disruptive influences are rare. However, two studies have yielded contrary results in this regard. Van Houwelingen et al. showed that experts in laparoscopic procedures exhibit fewer physical reactions than novices to disturbances during surgery. [22]. Moorthy et al. tested 12 surgeons with different levels of expertise (4 experts and 8 novices) on a laparoscopic trainer, using three levels of noise: quiet, 80–85 dB, and music. They tested the time taken to complete the exercise, the total number of movements, the total path length, and global scores. The authors registered no difference in the performance of experts and novices under the influence of noise or music [20]. Thus, it may be concluded that both, experts and novices must focus intensely on their surgical performance under disruption and would probably benefit from training with disruptive factors.

In the present study, persons who played a musical instrument in their leisure time did not perform better during disruption. The theory of transferring attention, concentration, and manual skills from the instrument to the operating room has been controversially discussed. Boyd et al. showed that playing an instrument improves laparoscopic skills in a laparoscopic suturing task [33], whereas Lin et al. and Yamacake et al. found that playing a musical instrument had no effect on a surgeon’s laparoscopic skills [13,14].

As three-dimensional orientation, reaction skills, and dexterity are required in laparoscopic and robotic-assisted surgery, the effect of video games on laparoscopic skills has been investigated quite extensively. A positive effect was noted in some studies [1,13,14]. However, a review of five randomized-controlled trials yielded no relevant difference [19]. In line with the latter report, we observed no relevant effect of video games on laparoscopic skills.

We registered no difference between the performance of women and men. Chiu et al. found that women performed better on a virtual simulator [34]. In another study addressing the gender-dependent performance of microsurgical skills, no difference was registered between women and men [35]. In view of the fact that only 29% of our participants were women, the comparison is not meaningful. However, it should be noted that 62% of all medical students in Germany are female [36], and women are gaining numerical predominance in the field of medicine. The present investigation shows once again that women should be given more opportunities for training in surgical specialties.

The self-estimation of the participants in regard of their skills correlated very strongly with the number of minimally invasive surgeries they had performed. No other factor, such as professional experience or experience in minimally invasive surgery showed such a strong correlation. The curve generated for this purpose approximated a classical learning curve [26]. As described in the introduction, uniform definitions do not exist for experts and novices in surgical disciplines. This makes it difficult to compare scientific studies. Furthermore, the classifications appear to be random and do not follow objective criteria. We lack uniform guidelines for the qualification of an expert in terms of clinical experience and certificates in surgery. Based on our learning curve, the required number of minimally invasive surgeries to achieve an expert level (>80% agreement) is between 500 and 1500 surgeries. However, our small number of cases and the wide range of data must be taken into account. Further studies in larger groups, with the inclusion of objective surgical skills, would narrow the range. Ranges for novices and mid-level experienced surgeons could also be specified in a large study population

In summary, neither experience, age, or personal skills transferred from leisure activities nor gender or even self-confidence based on expertise had an effect on the surgeons’ and novices’ performance of surgical tasks under disruption. None of those factors protected surgeons from distraction during surgery. Thus, a calm setting in the operating room appears to be crucial for every surgeon and for the safety of patients.

## 5. Conclusions

Interactive disruption is the most severe and common type of disturbance in operating rooms. It prolongs operating times, gives rise to errors, and affects the patient’s safety. Virtual reality training with disruption should be integrated into the education of surgeons. It would train surgeons to remain unaffected by disruption and focus on the patient. As disruption even affects the performance of experienced surgeons, any disturbance should be avoided in the operating room. This would pose a major challenge for logistics and personnel management. A combined strategy of training surgeons to perform surgery under disruptive conditions on the one hand and shielding the operating room from disruption on the other might be a feasible solution to the problem.

## Figures and Tables

**Table 1:** Experience of the participants.

	N	Mean (SD) %	Min./Max.	Median (IQR) %
Professional experience (years)	42	10.8 ± 11.3	0/52	9.5 (1.0–17.0)
Experience in minimally invasive surgery (years)	42	7.7 ± 11.3	0/37	4.5 (0–12.0)
Number of minimally invasive surgeries	42	524.8 ± 729.5	0/2500	23 (0–84)
Self-estimated experience in minimally invasive surgery as a surgeon (%)	42	39.6 ± 40.4	0/100	23 (0–84)
Self-estimated experience in practicing music (%)	42	34.5 ± 30.6	0/100	26 (11–59)
Self-estimated experience in video games (%)	42	37.2 ± 32.9	0/100	28 (5–65)

## Supplementary Materials

The following are available online at https://www.mdpi.com/1424-8220/20/20/5891/s1, Enclosure 1: Questionnaire completed during Peg Board in English. Enclosure 2: Questionnaire completed during Peg Board in German.
