# Supplementary material for: Impact of Acoustic and Interactive Disruptive Factors during Robot-Assisted Surgery—A Virtual Surgical Training Model
Source: Sensors (Basel). 2020 Oct 17;20(20):5891. doi: 10.3390/s20205891 (PMC7594064; doi:10.3390/s20205891)
Supplement: Supplementary file 1 [file sensors-20-05891-s001.pdf]

PROFESSIONAL AND PERSONAL QUALITIES OF PARTICIPANTS ON THE ROBOT SIMULATOR

Name: ____ **No.:** _______

Email: ____

Age: _________ **Male**: m / f / d

Professional experience: a) Resident ____

b) Specialist ____

c) Consultant ____

d) Clinical director ____

e) Others: ____________

Professional experience in years: _______

Experience in minimal invasive surgery in years: ______

How do you rate your expertise in laparoscopic surgery?

Novice *Expert*

a) As assistant ◆——————————◆

b) As surgeon ◆——————————◆

How many laparoscopic surgeries have you performed so far?

a) As assistant ________

b) As surgeon ________

How many laparoscopic operations do you perform per week?

a) As assistant ________

b) As surgeon ________

How do you rate your experience in the field of computer games?

Very low ◆——————————◆ *very high*

How do you rate your experience in practicing music?

Very low ◆——————————◆ *very high*

Handedness: a) right-handed

b) left-handed

c) ambidextrous

☐ I consent to the anonymous processing of my data and training results.

____

Signature

Thank you very much for your participation.

TEILNAHME ROBUSTHEITSCHECK AM ROBOTERSIMULATOR

Name, Vorname: ____ **Nr.:**_______

Emailadresse: ____

Alter: _________ **Geschlecht**: m / w / d

Stellenbezeichnung: a) Assistenzärztin/arzt ____

b) Fachärztin/arzt ____

c) Oberärztin/arzt ____

d) Chefärztin/arzt ____

e) Sonstige: ____________

Berufserfahrung in Jahren: _______

Erfahrung mit minimalinvasiver Chirurgie in Jahren: ______

Wie schätzen Sie Ihre Expertise in laparoskopischer Chirurgie ein? Bitte ankreuzen!

Anfänger *Experte*

a) als Assistenz ◆————————————————————◆

b) als Operateur ◆————————————————————◆

Wie viele laparoskopische Operationen haben Sie bisher in etwa durchgeführt

a) als Assistenz? ____

b) als Operateur? ____

Wie viele laparoskopische Operationen führen Sie pro Woche durch?

a) als Assistenz ____

b) als Operateur ____

Erfahrung im Bereich Computerspiele? Bitte ankreuzen!

Keine ◆————————————————————◆ *sehr viel*

Erfahrung im Praktizieren von Musik? Bitte ankreuzen!

Keine ◆————————————————————◆ *sehr viel*

Händigkeit: a) rechtshändig

b) linkshändig

c) beidhändig

☐ Ich bin mit der Weitergabe meiner Daten und Trainingsergebnisse in anonymisierter Form einverstanden.

____

Unterschrift

Herzlichen Dank für Ihre Teilnahme!
